# Brain structural correlates of subjective sleepiness and insomnia symptoms in shift workers

**DOI:** 10.3389/fnins.2024.1330695

**Published:** 2024-02-19

**Authors:** Hyunwoo Jeong, Hyewon Yeo, Kyung Hwa Lee, Nambeom Kim, Jiyoon Shin, Min Cheol Seo, Sehyun Jeon, Yu Jin Lee, Seog Ju Kim

**Affiliations:** ^1^Department of Education and Training, Yonsei University College of Medicine, Severance Hospital, Seoul, Republic of Korea; ^2^Department of Psychiatry, Sungkyunkwan University School of Medicine, Samsung Medical Center, Seoul, Republic of Korea; ^3^Department of Psychiatry and Center for Sleep and Chronobiology, Seoul National University Hospital, Seoul, Republic of Korea; ^4^Neuroscience Research Institute, Gachon University, Incheon, Republic of Korea

**Keywords:** shift worker, sleepiness, insomnia, brain imaging, neuroimaging, motor cortex, cortical surface area, putamen

## Abstract

**Background:**

Studies on the brain structures of shift workers are limited; thus, this cross-sectional study aimed to compare the brain structures and the brain structural correlates of subjective sleepiness and insomnia symptoms between shift workers and non-shift workers.

**Methods:**

Shift workers (*n* = 63) and non-shift workers (*n* = 58) completed questionnaires assessing subjective sleepiness and insomnia symptoms. Cortical thickness, cortical surface area, and subcortical volumes were measured by magnetic resonance imaging. The brain morphometric measures were compared between the groups, and interaction analyses using the brain morphometric measures as the dependent variable were performed to test the interactions between the study group and measures of sleep disturbance (i.e., subjective sleepiness and insomnia symptoms).

**Results:**

No differences in cortical thickness, cortical surface area, or subcortical volumes were detected between shift workers and non-shift workers. A single cluster in the left motor cortex showed a significant interaction between the study group and subjective sleepiness in the cortical surface area. The correlation between the left motor cortex surface area and the subjective sleepiness level was negative in shift workers and positive in non-shift workers. Significant interaction between the study group and insomnia symptoms was present for the left/right putamen volumes. The correlation between the left/right putamen volumes and insomnia symptom levels was positive in shift workers and negative in non-shift workers.

**Conclusion:**

Left motor cortex surface area and bilateral putamen volumes were unique structural correlates of subjective sleepiness and insomnia symptoms in shift workers, respectively.

## 1 Introduction

Approximately 20% of the working population worldwide is engaged in shift work (Alterman et al., 2013; Eurofound, 2017). Shift work disrupts the sleep–wake cycle and its synchrony with an individual’s circadian rhythms, leading to sleep disturbances, such as insomnia and excessive sleepiness (Wright et al., 2013). These sleep problems can harm the physical and mental health of shift workers (Wright et al., 2013; Brown et al., 2020). Shift workers can experience circadian rhythm misalignment due to conflicts between shift work schedules and the biological clock, which exacerbates sleep disturbances (James et al., 2017).

Previous investigations into the brain structures of shift workers have been limited and inconsistent. Two voxel-based morphometric studies have reported decreases in gray matter volume in several brain regions, including the postcentral gyrus, the paracentral lobule, the superior temporal gyrus, and the pontomesencephalic tegmentum (Kim and Kim, 2017; Park et al., 2020). However, a recent pilot study using surface-based analysis found no structural changes in the brains of either current or former shift workers (Bittner et al., 2022). Notably, the sizes of the samples from these three previous studies were relatively small; all of these studies recruited 20 or fewer current shift workers for analysis (Kim and Kim, 2017; Park et al., 2020; Bittner et al., 2022). In addition, one of these studies did not correct for multiple comparisons (Kim and Kim, 2017).

Different populations have different brain structural correlates of sleep-related measures. For example, subjective sleepiness correlates negatively with gray matter volume in the left gyrus rectus and the medial orbitofrontal cortex in healthy individuals (Killgore et al., 2012), while it is positively correlated with hippocampal volume in individuals with myotonic dystrophy type 1 (Hamilton et al., 2022). In addition, insomnia symptoms are positively correlated with cortical thickness in the right orbital frontal cortex and the fusiform area in individuals with primary insomnia (Yu et al., 2020), while insomnia symptoms are negatively associated with cortical surface area in several different brain regions in patients with major depressive disorder (Leerssen et al., 2020). Therefore, shift workers and non-shift workers may have different structural correlates of subjective sleepiness and insomnia symptoms. Some previous studies reported smaller gray matter volume in several brain areas in shift workers (Kim and Kim, 2017; Park et al., 2020), while another previous study did not find brain structural changes in shift workers (Bittner et al., 2022). However, no study has investigated the structural correlates of subjective sleepiness or insomnia symptoms in shift workers. Due to the paucity of conclusive findings, the present study compared the morphometric features of the brain (i.e., cortical thickness, cortical surface area, and subcortical volumes) between shift workers and non-shift workers in a relatively large sample. In addition, brain structural correlates of subjective sleepiness and insomnia symptoms were compared between shift workers and non-shift workers by interaction analyses. We hypothesized that brain structures would be altered in shift workers and that the relationship between brain morphometric measures and measures of sleep disturbance (i.e., subjective sleepiness and insomnia symptoms) would be different in shift workers and non-shift workers.

## 2 Methods

### 2.1 Participants

The participants were recruited from June 2017 to December 2019 via advertisements at Samsung Medical Center and Seoul National University Hospital. Shift workers who had either three-shift rotations of 8 h each or worked a shift beginning before 7 a.m. or ending after 6 p.m. were included. Only those who had worked as shift workers for more than 6 months were included. Non-shift workers included participants who did not engage in shift work, worked between 7 a.m. and 6 p.m., and did not have any sleep disturbances.

The Structured Clinical Interview for the Diagnostic and Statistical Manual of Mental Disorders-IV was conducted by trained psychologists to screen the participants for psychiatric disorders. Nocturnal polysomnography was performed to identify any common sleep disorders. Shift workers on the night shift were not allowed to undergo polysomnography during the day immediately following the nighttime shift. Exclusion criteria were: a history of serious medical or neurological illness; apnea-hypopnea index score ≥ 30 or periodic limb movement during sleep (PLMS) index score ≥ 50; Axis I psychiatric disorder other than a shift work type of circadian rhythm sleep disorder (based on the Diagnostic and Statistical Manual of Mental Disorders-IV); sleep disorder other than shift work sleep disorder (as defined by the International Classification of Sleep Disorders-3 criteria); pregnancy, and any contraindication for magnetic resonance imaging (MRI).

Initially, 65 shift workers and 64 non-shift workers were recruited. Of these, 2 non-shift workers were excluded because of sleep disorders. In addition, 1 shift worker and 1 non-shift worker were excluded due to anatomical abnormalities in their brain (e.g., tumor), and 1 shift worker and 3 non-shift worker whose gray/white matter boundaries could not be accurately drawn due to poor MRI quality were also excluded. Finally, 63 shift workers (15 men and 48 women; age 30.7 ± 6.6 years) and 58 non-shift workers (18 men and 40 women; age 31.6 ± 7.6 years) participated in this study. This study was approved by the Institutional Review Board of Seoul National University Hospital and was conducted following the Declaration of Helsinki. All participants provided written informed consent before participating in the study.

### 2.2 Measurements

Participants were asked to complete self-reported questionnaires at the start of the study. The Epworth Sleepiness Scale (ESS) was administered to assess subjective sleepiness by asking participants whether they were likely to fall asleep in eight different situations (Johns, 1991). Additionally, the Insomnia Severity Index (ISI) was employed to evaluate the severity of the nighttime and daytime components of insomnia (Morin et al., 2011). The levels of depressive and anxiety symptoms were assessed by the Beck Depression Inventory (BDI) and the Beck Anxiety Inventory (BAI), respectively (Beck et al., 1961, 1988). Participants compiled a sleep diary daily for 2 weeks to record their sleep-related information, and each participant’s average total sleep time was measured using this tool. Wakefulness after sleep onset (WASO) and apnea-hypopnea index (AHI) were measured by polysomnography to assess the degree of hyperarousal (Winkelman et al., 2008) and sleep apnea, respectively. Sleep efficiency (SE) and sleep onset latency (SOL) were also measured by polysomnography. Body mass index (BMI) was calculated to assess for obesity. The time of MRI scan was recorded for each participant.

### 2.3 Magnetic resonance imaging data acquisition

High-resolution structural imaging was obtained using a 3 T MRI system (Trio Tim; Siemens, Erlangen, Germany) with a 12-channel birdcage head coil. The 3D magnetization-prepared rapid-gradient echo scan was performed with the following parameters: Repetition time = 1,670 ms, echo time = 1.89 ms, inversion time = 900 ms, flip angle = 9°, slice thickness = 1.0 mm, in-plane resolution = 1.0 × 1.0 mm^2^, field-of-view = 250 mm, and matrix size = 256 × 256.

### 2.4 Data analyses

G*Power 3.1.9.2 software was used for sample size calculation. The minimum sample size yielded from this program was 90 (45 for group 1 and 45 for group 2). In the power analysis, an effect size of 0.6, power of 0.8 and α error probability of 0.05 were applied with a group allocation ratio of 1 (shift worker: non-shift worker = 1:1) based on the previous meta-analysis (Chang and Peng, 2021).

The demographic and clinical characteristics between the shift workers and non-shift workers were compared using the *t*-test and the chi-square test. Cortical reconstructions of the T1-weighted images from all study participants were accomplished using FreeSurfer software (version 7.1.1; http://surfer.nmr.mgh.harvard.edu; Dale et al., 1999). All coronal slices on all participants’ scans were visually inspected for accuracy of the gray/white matter boundary. The boundaries were manually edited as necessary, and scans were excluded from the study if edits were not possible because of poor image quality. Cortical thickness was measured by estimating the shortest distance between the white matter surface and the pial surface at each vertex, and cortical surface area was calculated as the average of the surrounding triangles at each vertex. The reconstructed brain of each participant was registered to a spherical space based on individual cortical folding patterns to match cortical geometry across participants, and each participant’s cortical thickness and cortical surface area measurements were assembled onto this new space. The cortical thickness and cortical surface area maps were smoothed with a full-width half-maximum Gaussian kernel of 10 mm before further analyses.

Cortical thickness and cortical surface area were compared between the two study groups using the general linear model (GLM) and corrected for multiple comparisons via nonparametric permutation cluster analysis (cluster-forming threshold: *p* < 0.05; cluster-wise value of *p*: *p* < 0.05, two-tailed). It is known that the cortical surface area scales with intracranial volume, whereas cortical thickness scales to a much lesser degree (FreeSurferWiki, 2023). Therefore, cortical thickness was compared between groups without any covariates, while the group comparison of cortical surface area was controlled for estimated total intracranial volume (eTIV).

Thalamus, caudate, putamen, pallidum, hippocampus, amygdala, and nucleus accumbens in both hemispheres were chosen for subcortical structure volume analysis, in line with previous studies that used FreeSurfer (Emamian et al., 2021; Gu et al., 2021). Segmentation of these 14 structures was performed in both hemispheres, and the volumes of these structures were measured using the automated procedure in FreeSurfer. Group comparisons of the subcortical structure volumes were performed by analysis of covariance (ANCOVA) correcting for eTIV. The 14 *p*-values derived from these analyses were adjusted for multiple comparisons using the false-discovery rate (FDR) method.

Interaction analyses via the GLM were performed using cortical thickness or cortical surface area as the dependent variable to test whether there were significant interactions between the study group and measures of sleep disturbance (i.e., ESS and ISI). The covariates for cortical thickness were age and sex, and the covariates for cortical surface area were age, sex, and eTIV. Multiple comparisons were corrected via nonparametric permutation cluster analysis (cluster-forming threshold: *p* < 0.05; cluster-wise value of *p*: *p* < 0.05, two-tailed). If the interaction analysis using cortical thickness as the dependent variable identified a cluster, a partial correlation between cortical thickness in the cluster and the sleep disturbance measure used in the analysis was calculated in each study group after adjusting for age and sex. Similarly, partial correlations were calculated between the cortical surface area and sleep disturbance measures after adjusting for age, sex, and eTIV for the cluster(s) showing significant interactions with cortical surface area.

Interaction analyses via multiple linear regression using each subcortical structure volume as the dependent variable were performed to test for significant interactions between the study group and measures of sleep disturbance (i.e., ESS and ISI). For each linear regression analysis, the dependent variable was the volume of the subcortical structure of interest, and the independent variables were age, sex, eTIV, study group, sleep disturbance measure (i.e., ESS and ISI), and study group × sleep disturbance measure interaction. The study group was encoded as a binary variable by assigning a value of 1 for shift workers and 0 for non-shift workers. As a result, 28 linear regression analyses were performed (14 subcortical structures × 2 sleep disturbance measures). The *p*-values of the interaction terms derived from the 28 regression models were adjusted for multiple comparisons using the FDR method. If one of these interaction analyses was significant, the partial correlation was calculated in each study group after adjusting for age, sex, and eTIV to investigate the relationship between the subcortical structure volume and the sleep disturbance measure used in the analysis.

Cortical reconstruction, preprocessing, and permutation cluster analysis were performed using FreeSurfer, and the remaining statistical analyses were performed using R software (version 4.0.2; The R Foundation for Statistical Computing, Vienna, Austria; R Core Team, 2014). A two-tailed value of *p* < 0.05 was considered significant.

## 3 Results

The demographic and clinical characteristics of the study participants are summarized in Table 1. The mean age and sex ratio did not differ significantly between shift workers and non-shift workers (30.7 ± 6.6 years vs. 31.6 ± 7.6 years, respectively; *p* = 0.52; 76.2% women vs. 69.0% women, respectively; *p* = 0.37). Among shift workers, the mean duration of such period of shift work was 66.2 ± 51.6 months. Most shift workers had rotating shifts (49.2% three-shift rotations and 28.6% two-shift rotations), and 4.8% had early morning shifts.

**Table 1:** Demographic and clinical characteristics of the study participants.

Variables	Shift workers (*n* = 63)	Non-shift workers (*n* = 58)	Value of *p*
Age (years)	30.7 (6.6)	31.6 (7.6)	0.52
Sex			0.37
Male (*n*) (%)	15 (23.8)	18 (31.0)	
Female (*n*) (%)	48 (76.2)	40 (69.0)	
Duration of shift work (months)	66.2 (51.6)	-	-
Shift work schedules			
Three-shift rotations (*n*) (%)	31 (49.2)	-	-
Two-shift rotations (*n*) (%)	18 (28.6)	-	-
Early morning shifts (*n*) (%)	3 (4.8)	-	-
Unclassified (*n*) (%)	11 (17.5)	-	-
ESS	9.2 (3.9)	7.7 (3.7)	0.03
ISI	9.6 (5.2)	8.0 (6.1)	0.12
BDI	8.1 (6.7)	6.3 (6.3)	0.13
BAI	7.5 (8.2)	7.8 (7.0)	0.84
Total sleep time (minutes)	400.8 (62.3)	411.4 (56.8)	0.42
WASO (minutes)	31.0 (43.3)	41.6 (41.6)	0.18
SE (%)	90.6 (10.4)	87.8 (11.2)	0.08
SOL (minutes)	8.6 (6.7)	13.3 (19.3)	0.00
AHI	1.6 (2.6)	2.2 (3.3)	0.27
BMI	21.2 (2.9)	22.1 (3.2)	0.10
Time of MRI scan	1:02 p.m. (105 min)	1:06 p.m. (109 min)	0.86

Continuous variables are presented as mean (standard deviation). ESS, Epworth Sleepiness Scale; ISI, Insomnia Severity Index; BDI, Beck Depression Inventory; BAI, Beck Anxiety Inventory; WASO, Wakefulness After Sleep Onset; SE, Sleep Efficiency; SOL, Sleep Onset Latency; AHI, Apnea-Hypopnea Index; BMI, Body Mass Index; MRI, Magnetic Resonance Imaging.

Shift workers had a higher ESS score than did non-shift workers (9.2 ± 3.9 vs. 7.7 ± 3.7, respectively; *p* = 0.03). Shorter SOL was shown in shift workers (8.6 ± 6.7) when compared to non-shift workers (13.3 ± 19.3). No significant differences in the ISI score (9.6 ± 5.2 vs. 8.0 ± 6.1, respectively; *p* = 0.12), BDI score (8.1 ± 6.7 vs. 6.3 ± 6.3, respectively; *p* = 0.13), BAI score (7.5 ± 8.2 vs. 7.8 ± 7.0, respectively; *p* = 0.84), total sleep time (400.8 ± 62.3 min vs. 411.4 ± 56.8 min, respectively; *p* = 0.42), WASO (31.0 ± 43.3 min vs. 41.6 ± 41.6 min, respectively; *p* = 0.18), SE (90.6 ± 10.4 vs. 87.8 ± 11.2), AHI (1.6 ± 2.6 vs. 2.2 ± 3.3, respectively; *p* = 0.27), or BMI (21.2 ± 2.9 vs. 22.1 ± 3.2, respectively; *p* = 0.10) were observed between shift workers and non-shift workers. The time of MRI scan did not differ between shift workers and non-shift workers (1:02 PM ± 105 min vs. 1:06 PM ± 109 min, respectively; *p* = 0.86).

Whole-brain analysis comparing cortical thickness and cortical surface area between shift workers and non-shift workers revealed no significantly different clusters. None of the 14 subcortical structures (i.e., left/right thalamus, caudate, putamen, pallidum, hippocampus, amygdala, or nucleus accumbens) had significant differences in volume between the two study groups.

**Figure 1:**
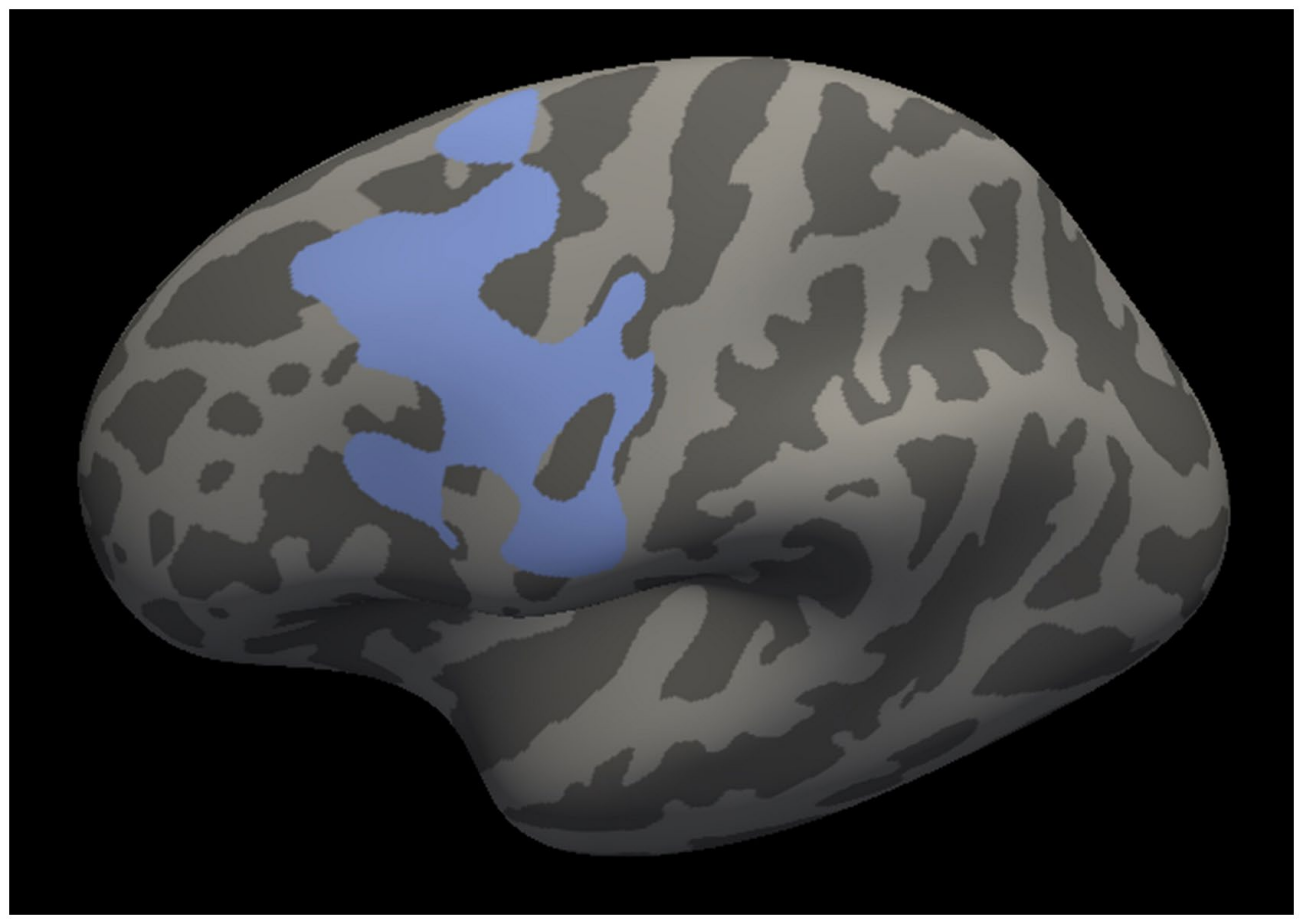
Left motor cluster in which the cortical surface area revealed a significant interaction between the study group and the Epworth Sleepiness Scale score. The cluster spans the primary motor cortex, the premotor cortex, and the supplementary motor area.

Whole-brain interaction analysis identified a single cluster in the left motor cortex in which the surface area showed significant interaction between the study group and the ESS score (cluster size = 3,282.1 mm^2^, cluster-wise *p* = 0.03; Figure 1). The cluster spanned the primary motor cortex, the premotor cortex, and the supplementary motor area. No other study group interaction with a sleep disturbance measure was found for cortical thickness or cortical surface area.

**Figure 2:**
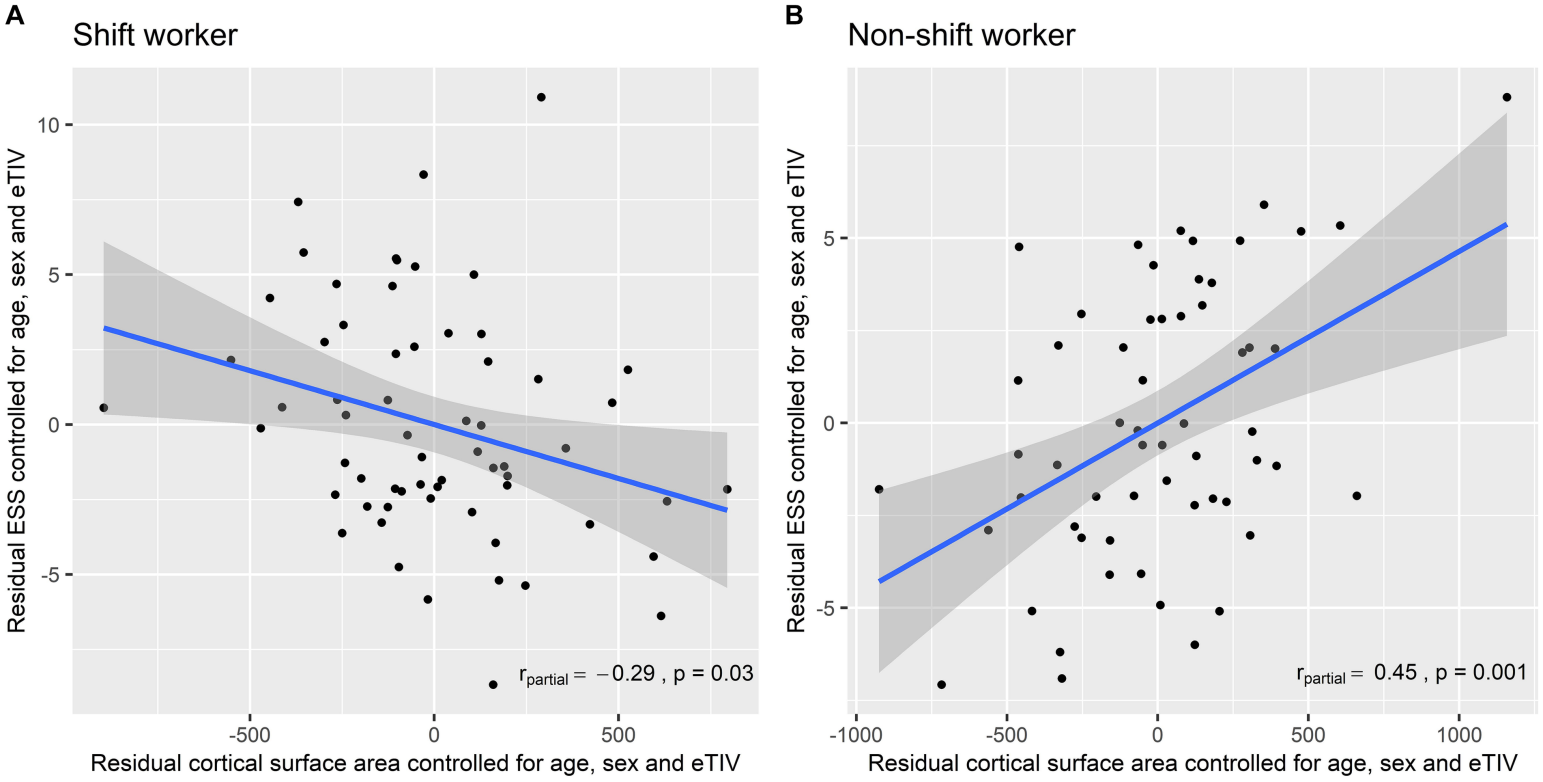
Partial correlation between the left motor cortex surface area and the Epworth Sleepiness Scale controlled for age, sex, and estimated total intracranial volume: **(A)** Shift workers; and **(B)** non-shift workers. ESS, Epworth Sleepiness Scale; eTIV, Estimated Total Intracranial Volume.

The associations between the cortical surface area of the left motor cluster and the ESS score in each group are presented in Figure 2. Among shift workers, the cortical surface area of the left motor cluster was negatively correlated with the ESS score after adjusting for age, sex, and eTIV (r_partial_ = −0.29, *p* = 0.03). In contrast, the partial correlation between the left motor cortex surface area and the ESS score was positive in non-shift workers (r_partial_ = 0.45, *p* = 0.001).

The results of the subcortical structure volume analyses exploring the interactions between sleep disturbance measures and the study group are presented in Table 2. Significant interactions between the study group and the ISI score were detected for left putamen volume (β = 0.55, FDR adjusted *p* = 0.02) and right putamen volume (β = 0.51, FDR adjusted *p* = 0.02). No other significant interactions were observed between the study groups and the sleep disturbance measures.

**Table 2:** Subcortical structural volume analysis investigating the interaction between the study group and measures of sleep disturbance.

Dependent variable: volume of subcortical structure	Interaction term					
	Study group × ESS			Study group × ISI		
	β	Unadjusted value of *p*	FDR adjusted value of *p*	β	Unadjusted value of *p*	FDR adjusted value of *p*
Left thalamus	0.14	0.45	0.84	0.21	0.13	0.68
Left caudate	−0.26	0.19	0.68	0.02	0.91	0.91
Left putamen	0.21	0.35	0.75	0.55^*^	0.001	0.02
Left pallidum	0.05	0.79	0.91	0.06	0.69	0.89
Left hippocampus	−0.08	0.70	0.89	−0.39	0.009	0.09
Left amygdala	0.13	0.52	0.85	−0.19	0.22	0.68
Left accumbens	0.03	0.88	0.91	0.04	0.78	0.91
Right thalamus	0.09	0.66	0.89	0.16	0.30	0.71
Right caudate	−0.14	0.50	0.85	0.02	0.88	0.91
Right putamen	0.26	0.22	0.68	0.51^*^	0.001	0.02
Right pallidum	0.10	0.63	0.89	0.19	0.26	0.68
Right hippocampus	−0.31	0.15	0.68	−0.24	0.15	0.68
Right amygdala	0.22	0.27	0.68	−0.11	0.44	0.84
Right accumbens	−0.11	0.63	0.89	−0.04	0.83	0.91

Independent variables: Age, sex, estimated total intracranial volume, study group, sleep disturbance measure (i.e., ESS and ISI), study group × sleep disturbance measure (interaction term). Study groups are encoded as follows: Shift workers: 1; non-shift workers: 0. ^*^FDR adjusted value of *p* < 0.05. ESS, Epworth Sleepiness Scale; ISI, Insomnia Severity Index; FDR, False Discovery Rate.

**Figure 3:**
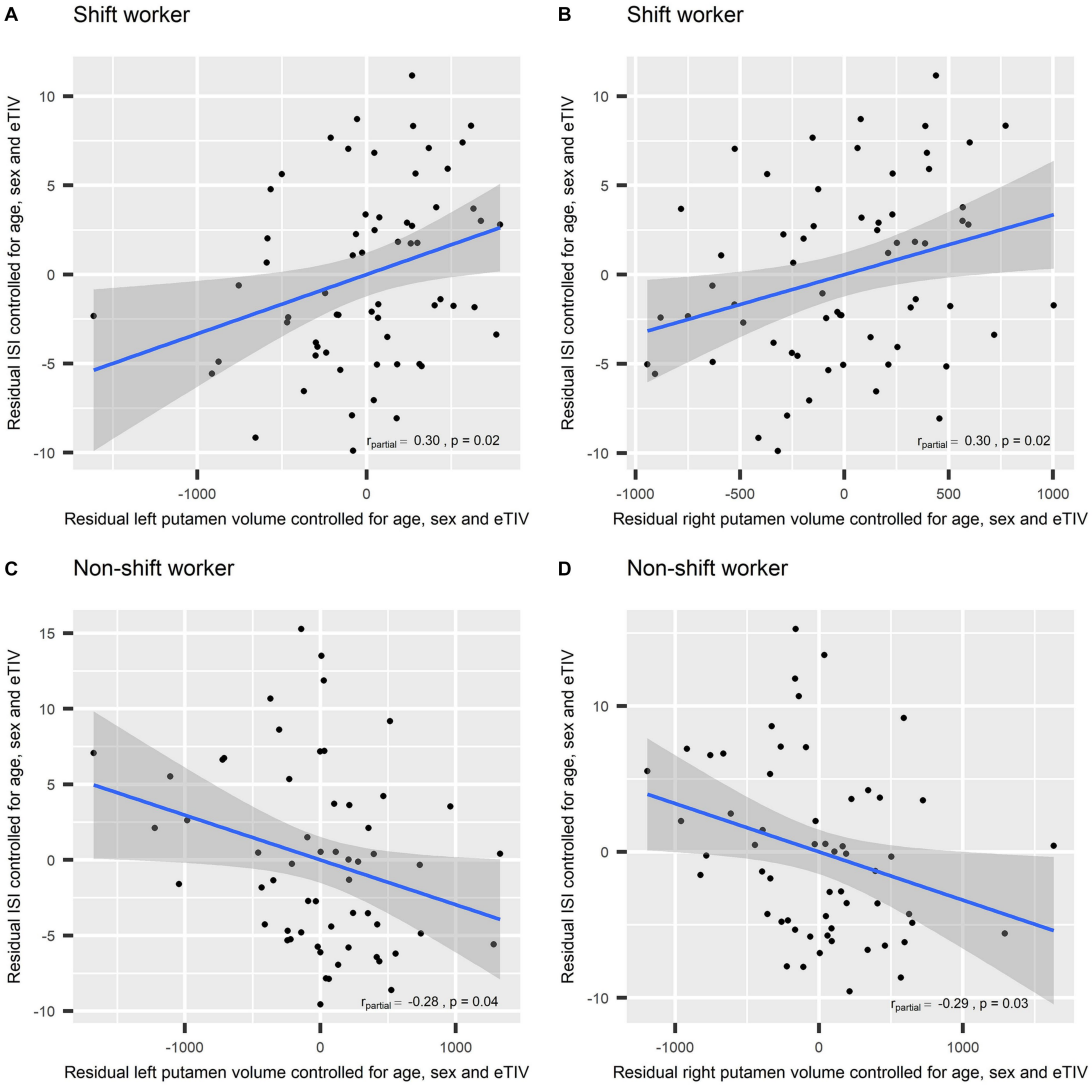
Partial correlation between left/right putamen volume and the Insomnia Severity Index controlled for age, sex, and estimated total intracranial volume: **(A)** Shift workers, left putamen; **(B)** shift workers, right putamen; **(C)** non-shift workers, left putamen; and **(D)** non-shift workers, right putamen. ISI, Insomnia Severity Index; eTIV, Estimated Total Intracranial Volume.

The associations between left/right putamen volume and the ISI score in each group are presented in Figure 3. Among shift workers, the volumes of the left and right putamen were positively correlated with the ISI score after adjusting for age, sex, and eTIV (r_partial_ = 0.30, *p* = 0.02; r_partial_ = 0.30, *p* = 0.02, respectively). In contrast, non-shift workers showed negative partial correlations between the volumes of the left and right putamen and the ISI score (r_partial_ = −0.28, *p* = 0.04; r_partial_ = −0.29, *p* = 0.03, respectively).

## 4 Discussion

The present study found no significant difference in cortical thickness, cortical surface area, or subcortical structure volume between shift workers and non-shift workers. However, unique structural correlates of subjective sleepiness and insomnia symptoms were discovered in shift workers; subjective sleepiness in shift workers negatively correlates with the cortical surface area of the left motor cortex, while their insomnia symptoms show a positive correlation with bilateral putamen volumes. Conversely, non-shift workers displayed associations in the opposite direction. To our knowledge, the present study is the first to report the brain structural correlates of subjective sleepiness and insomnia symptoms in shift workers.

In the present study, shift workers had higher levels of subjective sleepiness and sleep onset latency than non-shift workers, while total sleep time and the levels of insomnia, depressive, and anxiety symptoms were comparable between the two groups. The finding regarding subjective sleepiness is consistent with a previous study that demonstrated a high prevalence of excessive sleepiness in shift workers (Reis et al., 2020). However, in contrast to the findings of the present study, other studies reported short sleep duration and an increase in insomnia, depressive, and anxiety symptoms in shift workers (Kang et al., 2017; Voinescu, 2018; Chaiard et al., 2019). Notably, these studies did not specifically exclude participants with comorbidities (Kang et al., 2017; Voinescu, 2018; Chaiard et al., 2019), while the present study excluded participants who had psychiatric disorders or sleep disorders other than shift work sleep disorder. We speculate that the exclusion of comorbidities could explain the absence of shorter sleep duration or an increase in insomnia, depressive, or anxiety symptoms in our shift worker sample. This characteristic of our study population offered an opportunity to explore the effect of shift work itself by excluding the effect of other comorbid pathologies.

Hyperarousal, sleep apnea, and obesity are important factors related to subjective sleepiness and insomnia symptoms (Panossian and Veasey, 2012; Ong and Crawford, 2013; Kalmbach et al., 2018). Shift workers and non-shift workers had comparable levels of these factors in the present study. In addition, the time of imaging acquisition needs to be considered in brain structural analyses because brain volume is known to reduce from morning to evening (Trefler et al., 2016). The two study groups did not differ in the time of MRI scan. This shows that the present study’s findings were not affected by group differences in these relevant factors.

No brain structural differences were found between shift workers and non-shift workers in the present study. This finding is in line with a previous study that reported no changes in cortical thickness or subcortical volumes in shift workers (Bittner et al., 2022). On the other hand, another study reported that shift workers have reduced gray matter volume in several brain regions (Park et al., 2020). Importantly, shift workers had higher levels of depressive symptoms than non-shift workers in that study, and greater decreases in gray matter volumes were associated with higher depressive symptom levels (Park et al., 2020). This finding suggests that the shift workers’ gray matter volume could have been affected by their increased depressive symptoms, as depression is associated with structural changes in the brain (Kempton et al., 2011). The absence of significant group differences in the psychiatric symptom levels may explain why the brains of our shift worker sample were not structurally altered in contrast to the previous finding.

Significant associations between left motor cortex surface area and subjective sleepiness were found in the present study. Notably, a previous study reported that the functional connectivity between the thalamus and motor cortex was correlated with subjective sleepiness, suggesting disengagement of motor processing from the stream of consciousness during the sleepy state (Killgore et al., 2015). The present finding also supports the notion that the motor cortex is related to sleepiness.

It has been suggested that cortical surface area can be used to predict cortical excitability. One study revealed that the surface area of the visual cortex is positively correlated with the amplitude of the visual evoked potential, which is a measure of the cortical excitability of the visual cortex (Elvsåshagen et al., 2015). This finding implies that the cortical surface area of the motor cortex could be positively correlated with the cortical excitability of the corresponding brain region. Therefore, the negative correlation between the left motor cortex surface area and subjective sleepiness in shift workers may reflect a negative association between motor cortex excitability and subjective sleepiness, and the positive correlation in non-shift workers may reflect a positive association between motor cortex excitability and subjective sleepiness.

The fragmented sleep of shift workers could have led to a negative association between motor cortex excitability and subjective sleepiness. The circadian rhythms of shift workers often become misaligned with their work schedules (Kervezee et al., 2018), and this misalignment can lead to sleep fragmentation (Hasan et al., 2018). An experimental study reported that sleep fragmentation induces reduced motor cortex excitability and elevates the subjective sleepiness level (Scalise et al., 2014), suggesting an inverse relationship between motor cortex excitability and sleepiness in a sleep-fragmented state. This implies that the same inverse relationship could be present in shift workers, as sleep fragmentation is common in this population (Reid et al., 2018; Cheng and Drake, 2022), and may explain the negative correlation between the motor cortex surface area and subjective sleepiness among shift workers in the present study. Of note, similar findings were reported in individuals with obstructive sleep apnea (OSA); studies have revealed that OSA patients show decreased motor cortex excitability and increased daytime sleepiness (Grippo et al., 2005; Joo et al., 2010). Considering that OSA causes repeated end-apneic arousal during sleep (Kimoff, 1996), these findings may also be associated with sleep fragmentation.

On the other hand, sleep deprivation in non-shift workers could have led to a positive association between their motor cortex excitability and subjective sleepiness. The non-shift workers in the present study were largely deprived of sleep; their average total sleep time was 411 min per night. Considering that the appropriate sleep duration is 7–9 h for adults (Hirshkowitz et al., 2015), the average sleep duration of our non-shift worker sample was inadequate. Indeed, behaviorally induced insufficient sleep is very common among South Koreans (Park, 2020, 2022). According to the Organization for Economic Cooperation and Development (OECD), South Koreans sleep 41 min less per night than the average of the 38-member OECD nations (Park, 2022). Another survey revealed that 74.2% of South Korean workers suffer from sleep deprivation, and the reasons for their lack of sleep were mostly behavioral; the most common reasons included working late or spending time on the internet (Park, 2020). Studies have shown that a higher degree of sleep deprivation is associated with increased motor cortex excitability (Scalise et al., 2006; Huber et al., 2013) and greater sleepiness (Chokroverty, 2017), suggesting a positive association between motor cortex excitability and sleepiness in a sleep-deprived state. In line with these findings, a study that experimentally deprived healthy adults of sleep demonstrated a positive association between subjective sleepiness and cortical excitability in the supplementary motor area (Ly et al., 2016). Thus, under the sleep-deprived circumstance in South Korea, non-shift workers may have the same positive association between motor cortex excitability and subjective sleepiness. This may explain the positive correlation between motor cortical surface area and subjective sleepiness in non-shift workers in the present study. Notably, a previous study that investigated the association between gray matter volume and daytime sleepiness among young healthy individuals detected a positive correlation in the motor cortex within the left superior frontal gyrus, though the correlation became insignificant after correcting for multiple comparisons (Killgore et al., 2012).

Significant associations between putamen volume and insomnia symptoms were observed in the present study. The putamen promotes wakefulness by projecting inhibitory GABAergic neurons to the pallidum, which then projects to the cerebral cortex or the thalamus (mainly the mediodorsal nucleus; Qiu et al., 2010; Lazarus et al., 2013). In line with this finding, one study demonstrated that bilateral lesions of the putamen cause a reduction in the time spent awake (Qiu et al., 2010).

Evidence indicates that shift work could decrease the neuroplasticity of the putamen. The putamen is a known site of synaptic plasticity (Kreitzer and Malenka, 2008), and it has been reported that circadian rhythms affect the plasticity of putamen (Elmenhorst et al., 2016). Animal studies have demonstrated that simulated shift work impairs the synthesis of proteins related to synaptic plasticity (Marti et al., 2017, 2020). A decrease in the level of melatonin in shift workers (Wei et al., 2020) may contribute to the impaired neuroplasticity in this group, as melatonin is known to stimulate all stages of neuroplasticity (Valdés-Tovar et al., 2018).

The positive correlation between putamen volume and insomnia severity in shift workers could be explained by the low putamen plasticity in shift workers and the wake-promoting role of the putamen. As shift workers are expected to have little putamen plasticity, there would be little room for a compensatory change in the putamen in response to their sleep problems. In this case, shift workers with greater baseline putamen volumes may have more severe insomnia symptoms, given the wake-promoting role of the putamen. A similar positive association was also discovered between putamen volume and insomnia symptoms in older adults (Falgàs et al., 2021). Similar to shift workers, this finding may be explained by reduced synaptic plasticity of the putamen, as older age is associated with decreased striatal plasticity (Walsh and Akopian, 2019). Another analogy between shift workers and older adults is the disruption of circadian rhythms; older adults are prone to circadian desynchronization because early awakenings may result in frequent daytime naps and time cues for circadian rhythms (i.e., fixed work schedules or mealtimes) may be lacking due to retirement (Patel et al., 2018).

The negative correlation between putamen volume and insomnia severity in non-shift workers might be explained by the compensatory change in the putamen of non-shift workers in response to insomnia symptoms. Non-shift workers in the present study were anticipated to have great plasticity of the putamen, due to the absence of shift work and their young age. In this case, neuroplastic adaptation of the putamen could have occurred in response to insomnia symptoms; the putamen of non-shift workers with greater insomnia symptoms may undergo compensatory shrinkage to decrease arousal and promote sleep, leading to the negative association between putamen volume and insomnia severity. Two previous studies in relatively young adults reported a similar negative association between putamen volume and insomnia symptoms (Koo et al., 2017; Emamian et al., 2021). One study demonstrated that insomnia patients have smaller putamen volumes compared to controls (Emamian et al., 2021), and the other study showed that local volume loss of the putamen is associated with higher arousal indices among patients with chronic insomnia (Koo et al., 2017). These findings may also be explained by the same compensatory mechanism, as young adults are expected to have high putamen neuroplasticity.

This study had several limitations. First, causal relationships could not be determined as this study was cross-sectional; thus, longitudinal studies are required. Second, the small proportion of male participants may limit the generalizability of the current findings to other shift worker populations. Third, only self-reported two-week sleep diary was used in the current study. Self-report is more vulnerable for the recall bias, as individuals tend to retrospectively over- or under-estimate their experiences. Objective measures such as actigraphy would provide more unbiased evaluation for circadian rhythm. Fourth, the young age of the present study’s sample could be a limitation as the participants’ exposure to shift work was relatively short. However, previous studies have shown that structural plasticity can occur in short time scales (as early as within an hour) in the brain (Driemeyer et al., 2008; Xu et al., 2009). Fifth, no specific medication information was included in this analysis. However, as those with severe medical and mental illness were excluded from the study participants, the effect of medication may be not so large. Finally, subtle sleep fragmentation such as micro-arousal or EEG power changes was not assessed in the current study. These assessments would be helpful for investigating sleep fragmentation and its association with brain structures in shift workers.

In conclusion, no structural alterations were detected in the brains of shift workers, but the left motor cortex surface area and bilateral putamen volumes were their unique structural correlates of subjective sleepiness and insomnia symptoms, respectively. The negative association between the left motor cortex surface area and subjective sleepiness may be related to sleep fragmentation caused by shift work, and the positive association between putamen volumes and insomnia symptoms may be explained by decreased neuroplasticity due to shift work. Further research is needed to clarify the causal relationships and to explore the mechanisms behind the associations discovered here.

## Data availability statement

The raw data supporting the conclusions of this article will be made available by the authors, without undue reservation.

## Ethics statement

The studies involving human participants were reviewed and approved by the Institutional Review Board of Seoul National University Hospital. The participants provided their written informed consent to participate in this study.

## Author contributions

HJ: Formal analysis, Investigation, Methodology, Software, Visualization, Writing – original draft. HY: Formal analysis, Investigation, Methodology, Software, Writing – review & editing. KL: Data curation, Methodology, Resources, Writing – review & editing. NK: Data curation, Methodology, Resources, Software, Writing – review & editing. JS: Validation, Writing – review & editing. MS: Validation, Writing – review & editing. SJ: Validation, Writing – review & editing. YL: Conceptualization, Data curation, Funding acquisition, Methodology, Project administration, Resources, Software, Supervision, Validation, Writing – review & editing. SK: Conceptualization, Data curation, Funding acquisition, Methodology, Project administration, Resources, Software, Supervision, Validation, Writing – review & editing.
